# Molecular basis of host specificity in human pathogenic bacteria

**DOI:** 10.1038/emi.2014.23

**Published:** 2014-03-26

**Authors:** Xiaolei Pan, Yang Yang, Jing-Ren Zhang

**Affiliations:** Center for Infectious Disease Research, School of Medicine, Tsinghua University, Beijing 10084, China

**Keywords:** host specificity, immune evasion, molecular mechanisms, pathogen–host interactions, pathogenic bacteria, tropism

## Abstract

Pathogenic bacteria display various levels of host specificity or tropism. While many bacteria can infect a wide range of hosts, certain bacteria have strict host selectivity for humans as obligate human pathogens. Understanding the genetic and molecular basis of host specificity in pathogenic bacteria is important for understanding pathogenic mechanisms, developing better animal models and designing new strategies and therapeutics for the control of microbial diseases. The molecular mechanisms of bacterial host specificity are much less understood than those of viral pathogens, in part due to the complexity of the molecular composition and cellular structure of bacterial cells. However, important progress has been made in identifying and characterizing molecular determinants of bacterial host specificity in the last two decades. It is now clear that the host specificity of bacterial pathogens is determined by multiple molecular interactions between the pathogens and their hosts. Furthermore, certain basic principles regarding the host specificity of bacterial pathogens have emerged from the existing literature. This review focuses on selected human pathogenic bacteria and our current understanding of their host specificity.

## INTRODUCTION

Host specificity or tropism of a microbial pathogen is defined by its ability to colonize (or infect) a host organism.^1^ Pathogenic bacteria display various levels of host specificity. Certain bacteria have extremely diverse host ranges. Bacteria in this category may infect insects, humans, rodents and many other wild/domestic animals, such as *Borrelia burgdorferi* and *Yersinia pestis* (the respective etiologic agents of Lyme disease and plague). Many bacteria possess intermediate levels of host specificity. Such pathogens infect human and other mammalian species, but they may exhibit different pathogenicity between human and other hosts. For example, *Salmonella* Typhimurium causes gastroenteritis in humans after oral ingestion of the bacterium, but in mice, this infection route leads to symptoms resembling typhoid fever of human.^2,3^ *Listeria monocytogenes* can cause listeriosis in human and a number of domestic animals after oral ingestion of the bacterium. Interestingly, oral infection of *L. monocytogenes* leads to listeriosis-like disease in some model animals (e.g., guinea pig) but not other (e.g., mouse).^4,5,6^ These important features have made *S.* Typhimurium and *L. monocytogenes* important models for studying many important aspects of bacterial pathogenesis, host immunity, host-pathogen interactions, and host specificity.^2,3,7^ In sharp contrast, a number of other bacteria are highly adapted to the human environment and display strict host selectivity for humans, including *Haemophilus influenzae*, *Helicobacter pylori*, *Neisseria gonorrhoeae*, *Neisseria meningitidis*, *Mycobacterium leprae*, *Salmonella* Typhi, *Streptococcus pneumoniae*, *Streptococcus pyogenes*, *Vibrio cholerae* and *Treponema pallidum*. For the purpose of discussion in this review, we will hereafter refer to these bacteria as human-specific pathogens.

Understanding the genetic and molecular basis of host specificity in pathogenic bacteria is of great importance. First, learning how a pathogen determines its host range at the molecular level can directly enhance our knowledge of pathogenic mechanisms. Second, identifying the molecular determinants of host tropism for a pathogen provides insights that are valuable for the improvement of animal models used to simulate the human diseases caused by natural infection. Finally, defining the molecular mechanisms of bacterial host specificity can provide potential molecular targets and other clues to aid in the design of new strategies and therapeutics for the control of microbial diseases.

Host specificity of microbial pathogens is determined by elaborate molecular interactions between the pathogens and hosts. This principle is best demonstrated in viral pathogens due to the relative simplicity of their genomes and structures. The host specificity of viruses is predominantly defined by the interactions of viral proteins with their cognate cellular receptors.^8^ The molecular mechanisms of host specificity are less understood for bacterial pathogens due to the complexity of their molecular compositions and cellular structures. However, important progress has been made toward the complete understanding of the host specificity of pathogenic bacteria over the last two decades. This review focuses on the host specificity of selected human-specific pathogens. Due to space constraints, we will not provide exhaustive coverage of the discoveries in the area of bacterial host specificity. Instead, we will attempt to uncover emerging principles in this area by focusing on several bacterial pathogens for which the host specificity has been extensively characterized.

## HUMAN-SPECIFIC PATHOGENS

### Pathogenic *Neisseria*

*N. gonorrhoeae* and *N. meningitidis* are two human pathogens within the genus *Neisseria*. *N. gonorrhoeae* is the causative agent of gonorrhea (pelvic inflammation), a sexually transmitted disease. *N. meningitidis* causes invasive infections, such as septicemia and meningitis. Both pathogens have strict host tropism for humans. Like many other human-specific pathogens, the narrow host specificity of these pathogenic *Neisseria* has made it difficult to study the pathogenic mechanisms and develop therapeutics due to the lack of appropriate animal models that simulate the clinical presentations of the human diseases.^9,10,11^

The molecular basis for the host specificity of pathogenic *Neisseria* is not fully understood, but the existing evidence indicates that multiple factors are involved. On the pathogen side, a number of surface-exposed proteins appear to be associated with the human specificity of pathogenic *Neisseria*, including immunoglobulin A1 (IgA1) protease,^12,13^ type IV pili,^14,15,16^ complement factor H binding proteins (FHBP),^17,18^ gonococcal porin,^19^ transferrin-binding proteins^20,21^ and lactoferrin-binding proteins.^21,22^ On the host side, several human proteins have been implicated as host specificity determinants, including IgA1,^12,13^ cell surface complement regulator CD46,^10^ complement regulator factor H (FH),^17,18,23^ complement regulator C4b-binding protein (C4BP),^19^ transferrin^9,24,25^ and lactoferrin. ^24,25^

**Figure 1:**
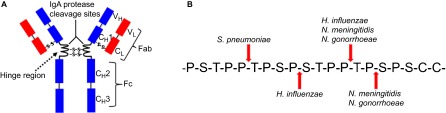
Cleavage of human IgA1 by bacterial IgA1 proteases. (**A**) Diagrammatic illustration of human IgA1. Indicated are the hinge region (target for bacterial IgA1 proteases) and the variable and constant regions of the IgA1 light (V_L_ and C_L_) and heavy (V_H_ and C_H_) chains. (**B**) Amino-acid sequence of the human IgA1 hinge region. The cleavage site for each of the bacterial IgA1 proteases is marked by the species name of the corresponding bacterium.

As illustrated in Figure 1, *N. gonorrhoeae* and *N. meningitidis* produce an extracellular serine-type protease that specifically cleaves human IgA1, a subclass of the dominant mucosal antibody, IgA.^12,13^ IgA1 is the most abundant antibody in upper respiratory secretions.^26^ IgA serves as a ‘non-inflammatory mucosal protector' at mucosal surfaces, performing multiple important functions, such as inhibition of microbial adherence, neutralization of microorganisms and inhibition of antigen penetration.^27^ *S. pneumoniae* and *H. influenzae*, two additional obligate human pathogens and colonizers of the human nasopharynx, possess similar capabilities to cleave human IgA1 (Figure 1).^13,28,29^ These IgA1 proteases specifically cleave the hinge region of IgA1, but not that of IgA2 due to the sequence differences of the two IgA subclasses in this region, with 26 amino acids in IgA1 and 13 in IgA2.^26^ Mouse and many other mammals possess only one IgA antibody with significant sequence differences from human IgA1, which explains why the IgA1 proteases of pathogenic *Neisseria* cannot digest the IgA produced by many nonhuman species.^30^ Cleavage of IgA1 in the hinge region leads to the separation of the Fc region from the antigen-binding Fab fragment, abolishing the crosslinking activity and all secondary effector functions mediated by the Fc region.^27^ The loss of IgA-mediated crosslinking and microbial agglutination may result in higher levels of bacterial adherence to mucosal surfaces and colonization.^13^

Type IV pili on the cell surface of pathogenic *Neisseria* mediate the initial adhesion to the mucosal epithelium through binding to human membrane cofactor protein (MCP or CD46).^14,15,16^ CD46 is a widely distributed cell membrane protein, which protects the host cells from accidental damage by complement through inactivating the alternative pathway of the complement system.^31,32^ The complement system is a key component of host immunity against *N. meningitidis*, and complement-deficient individuals have a significantly elevated risk of developing meningococcal disease.^33^ Previous studies have also identified CD46 as a host receptor for multiple viral and bacterial pathogens, such as measles virus,^34,35,36^ herpes virus 6,^37^ adenovirus^38,39,40,41^ and *S. pyogenes*.^42^ The extracellular segment of CD46 consists of four tandem complement control protein (CCP) modules and one or two heavily *O*-glycosylated serine/threonine/proline-rich domains.^43^ While measles virus and human herpesvirus 6 recognize the CCP1–2 and CCP2–3 modules, respectively,^44,45,46^ pathogenic *Neisseria* and *S. pyogenes* bind to the serine/threonine/proline-rich and CCP3–4 regions of CD46, respectively.^15,47^

Interaction of *N. meningitidis* and *N. gonorrhoeae* with CD46 triggers signaling events in the host cells, leading to intimate attachment and bacterial entry into host cells in cell culture models.^14,48,49,50^ The significance of this molecular interaction in the pathogenesis of *N. meningitidis* has been further implicated in transgenic mice expressing human CD46.^10^ When infected intraperitoneally with piliated *N. meningitidis*, CD46 transgenic mice displayed higher levels of bacteria in the bloodstream and cerebrospinal fluid; similar infection via intranasal inoculation with *N. meningitidis* also led to bacteremia and mortality only in CD46 transgenic mice, but not in the control mice. These *in vitro* and *in vivo* studies strongly suggest that CD46 is a host factor that contributes to the host specificity of *N. meningitidis*. Because the type IV pili of *N. gonorrhoeae* and *N. meningitidis* interact with CD46 in a similar manner,^14,48,49,50^ it is likely that this pathogen–host interaction also contributes to the host tropism of *N. gonorrhoeae* for humans. Transgenic mice expressing human CD46 are more susceptible to *S. pyogenes* infection (see below).^51,52^

**Figure 2:**
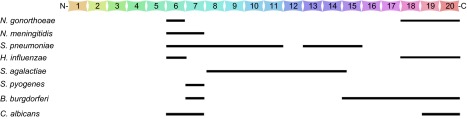
Schematic illustration of the molecular interactions between microbial pathogens and negative regulator protein factor H of the alternative complement pathway. The 20 FH SCR domains are illustrated in the context of their binding specificities for *N. gonorrhoeae*,^60,61^ *N. meningitidis*,^55,60^ *H. influenzae*,^62^ *S. pneumoniae*,^63,64,65^ *S. pyogenes*,^66,67^ *S. agalactiae*,^63^ *B. burgdorferi*^68,69^ and *C. albicans*.^70^ The information on the FH-binding activities was derived from the relevant references cited here for the corresponding microorganisms.

Both *N. gonorrhoeae* and *N. meningitidis* are able to recruit FH to the cell surface in a human-specific fashion.^53,54,55^ FH is one of the most abundant proteins in the blood (commonly present in human plasma at concentrations of 300 to 500 µg/mL). FH is composed of 20 short consensus repeats (SCRs), each containing approximately 60 amino acids.^56^ FH inhibits the alternative pathway of the complement system by preventing the binding of factor B to C3b, enhancing the decay of the C3-convertase (C3bBb) and acting as a cofactor for the cleavage of C3b by complement factor I.^57^ FH deposition on host tissue and cell surfaces prevents non-specific damage and avoids the wasteful consumption of complement components.^57^ Many microbial pathogens have been shown to bind to FH as a common mechanism for the evasion of complement-mediated host immunity (Figure 2).^58,59^ This is exemplified by the binding of FH to pathogenic *Neisseria*,^17,18,23,71^ *Candida albicans*,^70,72^ *Borrelia burgdorferi*,^68,69,73^ *H. influenzae*,^62^ *S. pneumoniae*,^63,74^ *S. pyogenes*,^75^ *S. agalactiae*^76,77,78^ and *Streptococcus suis*.^79^ Notably, *S. pneumoniae*, another resident of the human upper airway, also interacts with FH in a human-specific manner (see below).^80^

While *N. gonorrhoeae* binds to FH via its porin protein, a dominant component of the outer membrane,^17^ the FH binding activity of *N. meningitidis* has been localized to a ∼29-kD FHBP. FHBP is expressed as a lipoprotein in the meningococcal outer membrane of all strains of *N. meningitidis.*^18,81,82^ The recruitment of FH helps both pathogens evade complement-mediated lysis in human plasma.^17,18,23^ Moreover, FH-binding activity has been shown to mediate gonococcal attachment to human complement receptor 3 on the surface of epithelial cells, suggesting that this interaction may facilitate gonococcal entry into nonprofessional phagocytes and thereby aid the evasion of host immunity.^83^ The molecular preference of *N. gonorrhoeae* and *N. meningitidis* for human FH leads to a species-specific resistance to the bactericidal activity of human serum.^18,23,53,54^ Finally, the expression of human FH leads to significantly enhanced *N. meningitidis* bacteremia in transgenic rats.^84^ These findings are consistent with a clinical observation that polymorphisms in the promoter of the human FH gene are associated with increased risk for meningococcal disease in UK Caucasian patients.^85^

Prior to the discovery of its FH binding activity, the FHBP of *N. meningitidis* was identified as a vaccine candidate.^81,82^ Two FHBP-based vaccines have since advanced to clinical trials.^86^ The immunization of transgenic mice expressing human FH with the natural form of FHBP led to significantly decreased protective immune responses, as assessed by serum bactericidal activity; a mutant FHBP lacking the FH-binding activity showed the opposite vaccination result.^87,88^ This finding suggests that engineering FH binding-negative FHBP vaccine antigens may enhance the immunoprotective efficacy against meningococcal disease in humans.

*N. gonorrhoeae* and *N. meningitidis* bind to C4BP via the allelic variants of the porin protein.^19,89^ C4BP is a plasma glycoprotein that downregulates the classical complement pathway.^32^ It has been shown that *N. gonorrhoeae* binds only to human C4BP but not the counterparts of rodent, lagomorph and primate species.^53^ The species-specific binding pattern with human C4BP is consistent with gonococcal resistance to human serum complement.^53^ A number of other pathogens also recruit C4BP to evade complement-mediated immune responses, such as *Bordetella pertussis*,^90^ *Escherichia coli* K1,^91^ *H. influenzae*,^92^ *Moraxella catarrhalis*,^93^ *S. pneumoniae*^94,95^ and *S. pyogenes*.^96^ Interestingly, *S. pneumoniae*, also interacts with C4BP in a human-specific manner (see below).^94^

Iron acquisition appears to contribute to the host specificity of *N. gonorrhoeae* and *N. meningitidis* for humans ^25^. Iron is an essential nutrient for survival for virtually all bacteria. Pathogenic *Neisseria* species are able to extract iron from host iron-containing proteins through specific receptors, such as transferrin, lactoferrin and hemoglobin.^97^ *N. gonorrhoeae* and *N. meningitidis* preferentially bind to human transferrin and lactoferrin and utilize them as iron sources when compared with the transferrins and lactoferrins of other hosts.^25,98^ While the majority of transferrin circulates in human serum to deliver iron into cells and sequester free iron, lactoferrin is mostly present in phagocytic cells and in secretions such as milk, mucus and tears.^97^ Exogenous administration of human transferrin and lactoferrin through intravenous or intraperitoneal inoculation significantly enhances meningococcal bacteremia and mortality in mice.^24^ In addition, transgenic mice expressing human transferrin are significantly more susceptible to systemic infection by *N. meningitidis*.^9^ *N. meningitidis* utilizes human and bovine hemoglobins at a similar level *in vitro* and in mice,^24^ suggesting that the utilization of hemoglobin does not contribute significantly to the host specificity of pathogenic *Neisseria* for humans. In sharp contrast, the preferential binding of *Staphylococcus aureus* to human hemoglobin does contribute to its host specificity.^99^ Another interesting observation is that the human-specific binding of lactoferrin by pneumococci is not a means of iron acquisition ^100^. These lines of evidence indicate that different pathogens interact with host-containing proteins for various purposes.

### *Streptococcus pneumoniae*

*S. pneumoniae* is an obligate human pathogen and causes numerous infections, such as pneumonia, otitis media, meningitis and sinusitis in humans.^101^ The molecular mechanisms behind this strict host specificity are unclear, but several human-specific pathogen–host interactions have been recently revealed using biochemical approaches.

The metallo-type IgA1 protease of *S. pneumoniae* cleaves the hinge region of human IgA1 but not those of the IgA molecules from many other mammalian species (Figure 1).^28,29,30^ As discussed above, the digestion of IgA1 by bacterial IgA1 proteases leads to the loss of the crosslinking activity and all secondary effector functions mediated by the Fc region.^27^ *S. pneumoniae* IgA1 protease has been shown to enhance bacterial adherence to epithelial cells by digesting IgA1,^102^ suggesting that this human-specific activity is a host specificity determinant of *S. pneumoniae*.

*S. pneumoniae* interacts with a list of host factors in a human-specific manner through choline-binding protein A (CbpA). CbpA, also called PspC,^103^ is a major surface-exposed protein of *S. pneumoniae*.^104^ CbpA binds to the polymeric immunoglobulin receptor (pIgR),^105^ secretory component (SC),^106^ secretory IgA (SIgA),^106^ FH,^107,108^ C4BP,^95^ sialic acid,^104^ complement C3 protein^109^ and vitronectin.^110^ The middle repeat region of CbpA is responsible for binding to domains 3 and 4 of human pIgR and the same region in human SC and SIgA.^105,111,112,113^ While the FH binding activity has been mapped to the amino terminal region of CbpA and its allelic variants,^77,107,114^ multiple regions of human FH have been reported to bind to CbpA, including SCR 6–10,^65^ 8–14^63^ and 13–15.^64^ The CbpA–pIgR interaction mediates the epithelial adhesion and transmigration of *S. pneumoniae*;^105,115,116,117^ the recruitment of FH to the pneumococcal surface enhances the evasion of complement-mediated phagocytosis^74,80,107^ and epithelial invasion.^118^ Studies from our laboratory and others have demonstrated that CbpA exclusively binds to the pIgR, SC, SIgA and FH of humans but not their counterparts in common model animals (e.g., mouse, rat and rabbit).^80,105,112,119^ These species-specific interactions strongly suggest that CbpA is a major molecular determinant of pneumococcal host specificity.

The enolase of *S. pneumoniae* binds to C4BP, a negative regulator of the classical complement pathway.^94^ This interaction leads to decreased C3b deposition on pneumococci. Interestingly, this binding occurs only with human C4BP, not mouse C4BP.^94^ This observation is reminiscent of the human-specific recruitment of C4BP by *N. gonorrhoeae*, a colonizer of mucosal surfaces in humans.^53^ Thus, evasion of complement-mediated immunity appears to be a common mechanism underlying the host specificity of many pathogenic bacteria that colonize the mucosal surfaces of humans.

Pneumococcal surface protein A (PspA) is another protein that may contribute to the host tropism of *S. pneumoniae*. PspA is a major surface-exposed protein and a protective antigen of *S. pneumoniae*.^120^ Previous biochemical studies have shown that the C-terminal region of PspA binds to human lactoferrin, an iron-sequestering glycoprotein (predominately in mucosal secretions).^121^ Unlike pathogenic *Neisseria*, *S. pneumoniae* cannot obtain iron from lactoferrin.^122^ Instead, PspA binding to lactoferrin protects pneumococci from killing by apolactoferrin, a form of lactoferrin that does not carry iron.^123,124^ Interestingly, this interaction is human specific because PspA does not bind to bovine lactoferrin,^100^ suggesting that the PspA–lactoferrin interaction contributes to the host specificity of *S. pneumoniae*.

These species-specific pathogen–host interactions correlate very well with the host specificity of *S. pneumoniae*. It is thus tempting to conclude that host specificity of *S. pneumoniae* is defined by multiple molecular determinants. Among the bacterial factors, CbpA mediates multiple molecular species-specific interactions with human factors (e.g., pIgR, SC, SIgA and FH). The lack of appropriate animal models has been a major challenge for determining the biological impacts of these pathogen–host interactions on the host specificity and pathogenesis of *S. pneumoniae*. However, as exemplified in *L. monocytogenes*,^125,126^ these biochemical findings have provided critical molecular targets to establish genetically modified (humanized) animals and/or (murinized) pneumococci. These tools will be important for further elucidating the mechanisms of pneumococcal host specificity and evaluating new therapeutics and vaccines for the control of pneumococcal disease.

### *Streptococcus pyogenes*

*S. pyogenes* (group A streptococcus) is an obligate human pathogen that causes pharyngitis (strep throat), localized skin infections, scarlet fever, streptococcal toxic shock syndrome and autoimmune-mediated complications.^127^ Although the molecular basis for the human specificity of *S. pyogenes* remains unclear, a study reported by Sun *et al.*^128^ indicates that the streptokinase secreted by this bacterium is an important determinant of its host specificity. Streptokinase is able to bind to and activate human plasminogen, a blood clot-dissolving protein, but not the plasminogens from other mammalian species.^129^ The plasminogen-activating activity of streptokinase has been implicated in the accelerated clearance of host fibrin, which may promote the dissemination of *S. pyogenes* in host tissues.^128,130^ Mice typically possess high resistance to skin infection by *S. pyogenes*, but Sun *et al*.^128^ showed that the expression of human plasminogen in mice led to markedly increased mortality in the transgenic mice when infected with streptococci.

The molecular interaction of M protein with CD46 also contributes to the host specificity of *S. pyogenes*.^42^ The M protein of *S. pyogenes* is a major surface-exposed protein and one of its most important virulence factors.^131^ The specific interaction between M protein and human CD46 mediates streptococcal binding to keratinocytes ^42,132^ and the bacterial invasion of epithelial cells.^133^ Transgenic mice expressing human CD46 are more susceptible to streptococcal disease.^51,52^ In a systemic infection model, transgenic mice expressing human CD46 displayed higher levels of bacteremia, arthritis and mortality compared with the non-transgenic mice.^51^ Matsui *et al*.^52^ corroborated this finding in a subcutaneous infection model, in which the CD46 transgenic mice exhibited more severe forms of bacterial growth in deep tissues, necrotizing fasciitis at the infection sites (footpads) and mortality. These studies indicate that M protein is a molecular determinant of the host specificity of *S. pyogenes* through the targeting of human CD46.

*S. pyogenes* M protein also interacts with a wide range of other host factors, such as the complement regulator proteins C4BP^134^ and FH,^75^ as well as fibrinogen^135,136^ and IgG.^137,138^ *S. pyogenes* interactions with C4BP and FH are associated with bacterial evasion of complement-mediated immunity.^130^ It remains to be determined whether these pathogen–host interactions also contribute to the host specificity of this pathogen.

## OTHER PATHOGENIC BACTERIA

### Gram-positive bacteria

Host specificity has been studied to various extents in other pathogenic bacteria. Among them, *L. monocytogenes* is the best-characterized bacterial pathogen in terms of our knowledge of the molecular mechanisms of its host specificity. *L. monocytogenes* is a facultative intracellular bacterium that causes foodborne diseases in humans and domestic animals. Infection occurs when ingested bacteria cross the intestinal barrier and disseminate to various organs and the bloodstream, leading to listeriosis. However, many laboratory animals do not develop typical listeriosis through the oral ingestion of *L. monocytogenes*, such as mouse, rat, rabbit and guinea pig.^4,5,6^ Previous studies have revealed multiple molecular interactions that can now explain the host restriction of *L. monocytogenes*, as discussed in several excellent reviews.^125,139,140^

The host selectivity of *L. monocytogenes* depends on two surface-exposed bacterial proteins, internalin A (InlA) and internalin B (InlB). InlA and InlB promote the entry of *L. monocytogenes* into nonphagocytic cells by interacting with their cognate cellular receptors. InlA binds to E-cadherin, a major cell–cell adhesion glycoprotein;^141,142^ this interaction occurs with human E-cadherin but not the mouse counterpart due to a single amino-acid difference between the human and mouse E-cadherin proteins.^143^ The importance of the InlA–E-cadherin interaction to the host specificity of *L. monocytogenes* has been demonstrated in mice expressing human E-cadherin,^144^ ‘humanized' mouse E-cadherin^6^ and the ‘murinized' InlA of *L. monocytogenes*.^126^ The local expression of human E-cadherin in the small intestine of mice was shown to lead to InlA-dependent bacterial invasion of enterocytes, dissemination across the intestinal barrier and significantly higher mortality following oral infection of the transgenic mice with *L. monocytogenes*.^144^ Based on the structural knowledge of the InlA–human E-cadherin complex,^141^ Wollert *et al.*^126^ recently constructed a recombinant InlA that could interact with mouse E-cadherin by introducing two sequence substitutions in InlA, leading to dramatic enhancement in its binding affinity for mouse E-cadherin. The ‘murinized' *L. monocytogenes* expressing the engineered InlA was able to induce typical listeriosis in wild-type mice by oral infection.^126^

InlB is also a host specificity determinant of *L. monocytogenes*.^5^ It is essential for the entry of *L. monocytogenes* into hepatocytes and some epithelial and fibroblast cell lines.^145,146,147^ Three different host receptors have been reported to interact with InlB: C1q-binding protein gC1q-R/p32,^148^ Met (the receptor for hepatocyte growth factor)^149^ and glycosaminoglycans.^150^ InlB interacts with Met in a species-specific manner because it binds to the Met proteins of human and mouse but not those of rabbit and guinea pig.^5^ Consistently, only the Met proteins from human and mouse support the InlB-dependent entry of *L. monocytogenes* into mammalian cells; the InlB-deficient mutant does not show attenuated virulence in guinea pigs and rabbits. Among the common model animals, the gerbil is the only species in which both InlA/E–cadherin and InlB–Met interactions are functional.^6^ In agreement, the oral infection of gerbils with *L. monocytogenes* leads to typical listeriosis.^6,151^ These advancements have not only explained the pathogenic mechanisms of *L. monocytogenes*, but have also laid a new foundation for the rational design of better small animal models in studying human infections caused by this pathogen.^151^

*S. aureus* captures hemoglobin for heme-iron acquisition through the hemoglobin-binding protein IsdB.^152^ Pishchany *et al.*^99^ showed preferential binding of *S. aureus* to human hemoglobin when compared with murine hemoglobin. Consistently, human hemoglobin-expressing transgenic mice were significantly more susceptible to systemic infection by *S. aureus*, suggesting that iron availability in human contributes to the host specificity of *S. aureus.*^99^ Preferential binding to human hemoglobin is also observed with other primarily human-associated bacteria, including *Staphylococcus lugdunensis*, *Staphylococcus simulans* and *Corynebacterium diphtheriae*.^99^

### Gram-negative bacteria

*Salmonella enterica* can cause a wide spectrum of diseases in many hosts. Different serovars of *S. enterica* display either a restricted host range (e.g., *S.* Typhi for human, *S*. Dublin for cattle, swine and human) or a broad host range (e.g., *S*. Enteritidis and *S*. Typhimurium for human and many other hosts). The mechanisms of *Salmonella* host adaptation and specificity have been exclusively discussed in two recent excellent reviews.^2,3^ The major factors that affect the host specificity of *Salmonella* serovars include differences in the bacteria themselves (e.g., the ability to survive host immunity, to grow in a given host environment, and to transmit to other individuals of the same host species) and host environments (e.g., pH, temperature, immune recognition and response, and the microbiota of a given host).^153,154^

Pathogenic *E. coli* strains can cause numerous intestinal or extraintestinal infections. *E. coli* strains from human and domestic animals/birds often display host specificity.^155^ The host specificity of pathogenic *E. coli* can be influenced by the molecular interactions of bacterial surface-exposed proteins with host cellular receptors.^155,156,157,158^ Septicemic *E. coli* strains from chickens and lambs express host-specific adhesins, such as avian-specific AC/I pili and lamb-specific K99 fimbriae.^155,156,157^ Stromberg *et al*.^158^ have demonstrated that the distinct recognition of Galα 1-4Gal-containing glycolipid receptors on host cells contributes to the host specificity of uropathogenic *E. coli*.

## EMERGING PRINCIPLES

**Figure 3:**
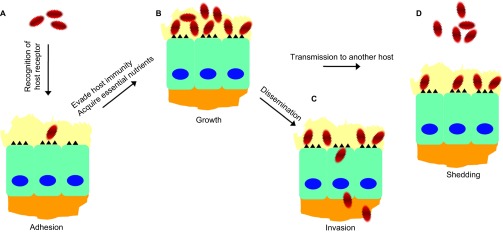
Major requirements for successful infection of human hosts by pathogenic bacteria. (**A**) Once bacteria enter the host, they need to adhere to mucosal surfaces by recognizing specific receptors on host cells. (**B**) The bacteria must be able to evade host immune mechanisms for survival (e.g., complement system, antibody) and acquire the necessary nutrients for the growth and expansion of the population (e.g., iron). (**C**) Once the population size has expanded to a certain level, bacteria can disseminate to other tissues/organs from the initial infection site by intracellular invasion through molecular interactions with host receptors or other means. (**D**) Some bacteria may be released into the surrounding environments and transmitted to another human host.

In contrast to viruses, bacteria clearly depend on much more complex molecular interactions with their natural hosts to determine host specificity. For this reason, the existing information is not sufficient to paint a full picture for the molecular mechanisms of bacterial host specificity, even for the most extensively studied bacterial pathogens (e.g., *L. monocytogenes* and pathogenic *Neisseria*). However, several principles have emerged from previous studies. As illustrated in Figure 3, the strong selectivity for humans of bacterial pathogens can be explained by the following: (i) specific recognition of a human receptor by pathogens for colonization and/or dissemination; (ii) specialized ability to evade or overcome immune mechanism(s) for bacterial survival; and (iii) availability of an essential nutrient for bacterial growth in humans.

### Colonization and dissemination

Certain surface-associated or secreted molecules contribute to host specificity by promoting bacterial colonization and/or dissemination. As well-characterized examples for bacterial adhesion and colonization, the pili of pathogenic *Neisseria*^14,15,16^ and *E. coli*^155,156,157,158^ mediate bacterial adherence to mucosal epithelia in a host-specific manner. The surface proteins of *S. pneumoniae* (CbpA) and *S. pyogenes* (M protein) bind to their human receptors for mucosal adhesion and dissemination.^42,105^ The surface-exposed proteins InlA and InlB of *L. monocytogenes* promote bacterial invasion across cellular/tissue barriers for dissemination into remote sites (Table 1).^5^ The streptokinase secreted by *S. pyogenes* promotes bacterial dissemination by fibrinolysis through interacting with human plasminogen.^129^

### Immune evasion

Evasion of host immune mechanisms, particularly complement- and IgA1-mediated immunity, is a common mechanism underlying the host specificity of many mucosal colonizers in humans. This is exemplified by the host-specific interactions with human C4BP, a negative regulator of the classical complement pathway, of many bacteria such as *N. gonorrhoeae*,^19^ *N. meningitidis*,^89^ *S. pyogenes*^134^ and *S. pneumoniae*.^94,95^ Similarly, many pathogens interact with negative regulators of the alternative complement pathway CD46 ^14,42^ and FH^53,54,80^ in a human-specific manner. Overcoming human IgA1-mediated immunity by bacterial proteases is a common mechanism underlying the host specificity of bacteria that naturally colonize the mucosal surfaces of the reproductive tract (e.g., *N. gonorrhoeae*) or the upper airway (e.g., *S. pneumoniae*, *N. meningitidis* and *H. influenzae*) (Table 1).^13^ These lines of evidence imply that complement- and IgA-mediated immunity is crucial for host defense against these pathogens in non-permissive hosts other than humans.

### Nutrient acquisition

Bacterial preference for host-specific nutrients is another common mechanism underlying host specificity. Although the precise nutrient requirements of individual bacteria in their hosts are complex, iron is one essential nutrient for bacterial growth *in vivo*.^97^ Many bacteria acquire iron from host iron-containing proteins by expressing specific receptors for these host proteins.^97^ Thus, preferential interactions of pathogenic bacteria with human iron-containing proteins have been demonstrated to contribute to the host specificity of *N. meningitidis* (transferrin and lactoferrin),^159^ *N. gonorrhoeae* (transferrin)^159^ and *S. aureus* (hemoglobin) (Table 1).^99,152^ Similarly, PspA-mediated binding to lactoferrin contributes to the human tropism of *S. pneumoniae*, although this notion remains to be validated by *in vivo* studies.

## PERSPECTIVES

Recent technologic advancements in the transgenic expression of human genes in model animals have led to the generation of many ‘humanized' mouse strains for the study of human-specific infectious diseases.^160^ As discussed above, some of the transgenic animals have been used to evaluate the contributions of species-specific biochemical interactions to the host specificity of individual bacterial pathogens. These include mouse strains expressing human E-cadherin for *L. monocy*togenes,^144^ human plasminogen for *S. pyogenes*,^128^ human CD46 for *N. meningitidis*^10^ and *S. pyogenes*,^51,52^ human transferrin for *N. meningitidis*,^9^ and rats expressing human FH for *N. meningitidis*.^84^ Furthermore, as exemplified in *L. monocytogenes*,^126^ the increasing availability of structural information on many pathogen–host molecular interactions has made it possible to engineer murinized bacterial strains that can simulate human disease in mice. Therefore, the revelations regarding species-specific pathogen–host interactions in previous studies have already established a foundation for the establishment of genetically engineered animals and bacterial strains for studying the host specificity and pathogenesis of obligate human pathogens in the future.

Finally, insightful information on the molecular mechanisms underlying bacterial host specificity may be applied to the control of human infections caused by bacterial pathogens. For example, the host specificity determinants of bacterial pathogens may be targeted for therapeutic and vaccine development in the future because these proteins tend to be highly conserved within individual bacteria. This is exemplified by the recent development of meningococcal vaccines based on the FH-binding protein of *N. meningitidis*. In this case, selectively inactivating the FH-binding activity of the FH-binding protein led to enhanced immunoprotective efficacy of the vaccine.^86,87,88^ Because the CbpA, PspA and IgA1 proteases are among the protein antigens that are immunoprotective against *S. pneumoniae* infections,^161,162^ selective mutations in the functional sites of these proteins may be used to improve the protective efficacies of these proteins as vaccine candidates.

## Figures and Tables

**Table 1:** Species-specific molecular interactions between bacteria and hosts

Bacterium	Bacterial ligand^a^	Host target molecule^b^	Natural host	Function	Reference
*N. gonorrhoeae*	IgA1 protease	IgA1	Human	Immune evasion	12, 13
	Porin protein	FH	Human	Immune evasion	61
	Type IV pili	CD46	Human	Adhesion/colonization; invasion/dissemination	10
	Porin protein	C4BP	Human	Immune evasion	53
	TbpA, TbpB	Transferrin	Human	Nutrient acquisition	25
	LbpA, LbpB	Lactoferrin	Human	Nutrient acquisition	25
*N. meningitidis*	IgA1 protease	IgA1	Human	Immune evasion	12, 13
	FHBP	FH	Human	Immune evasion	54
	Porin protein	C4BP	Human	Immune evasion	89
	Type IV pili	CD46	Human	Adhesion/colonization; invasion/dissemination	10
	TbpA, TbpB	Transferrin	Human	Nutrient acquisition	20, 24
	LbpA, LbpB	Lactoferrin	Human	Nutrient acquisition	20, 24
*H. influenzae*	IgA1 protease	IgA1	Human	Immune evasion	13, 29
*E. coli*	P-Fimbriae	Glycolipsids	Human, domestic animals	Adhesion/colonization	158
*L. monocytogenes*	InlA	E-cadherin	Human, domestic animals	Invasion/dissemination	6, 126, 143
	InlB	Met, gC1q-R/p32, glycosaminoglycans	Human, domestic animals	Invasion/dissemination	5, 6
*S. pneumoniae*	IgA1 protease	IgA1	Human	Immune evasion	13, 29
	CbpA	FH	Human	Immune evasion	80
	CbpA	pIgR/SC/SIgA	Human	Adhesion/colonization	106, 112
	Enolase	C4BP	Human	Immune evasion	94
	PspA	Lactoferrin	Human	Nutrient acquisition	100, 121
*S. pyogenes*	Streptokinase	Plasminogen	Human	Invasion/dissemination	128
	M protein	CD46	Human	Adhesion/colonization	42, 51, 52
*S. aureus*	IsdB	Hemoglobin	Human	Nutrient acquisition	99, 152

a Bacterial proteins that recognize host target molecules.

b Host molecules that are specifically recognized by bacterial factors.
